# Predictors of maternal health services uptake in West African region: a multilevel multinomial regression analysis of demographic health survey reports

**DOI:** 10.1186/s12978-024-01782-5

**Published:** 2024-04-06

**Authors:** Aklilu Habte, Samuel Hailegebreal, Atsedu Endale Simegn

**Affiliations:** 1 School of Public Health, College of Medicine and Health Sciences, Wachemo University, Hosanna, Ethiopia; 2 College of Medicine and Health Sciences, School of Public Health, Department of Health Informatics, Wachemo University, Hosaena, Ethiopia; 3 Department of Anesthesia, Wachemo University, Hosaena, Ethiopia

**Keywords:** Maternal health services, Multilevel, Adequate, Partial, Predictors, West Africa

## Abstract

**Background:**

Pursuant to studies, receiving the three key maternal health services (Antenatal Care, Skilled Delivery Service, and Postnatal Care) in a continuum could prevent 71% of global maternal deaths. Despite the Western African region being known for its high maternal death and poor access to maternal health services, there is a dearth of studies that delve into the spectrum of maternal health services uptake. Hence, this study aimed to assess the level and predictors of partial and adequate utilization of health services in a single analytical model using the most recent Demographic and Health Survey (DHS) data (2013–2021).

**Methods:**

This study was based on the appended women's (IR) file of twelve West African countries. STATA software version 16 was used to analyze a weighted sample of 89,504 women aged 15–49 years. A composite index of maternal health service utilization has been created by combining three key health services and categorizing them into ‘no’, ‘partial’, or ‘adequate’ use. A multilevel multivariable multinomial logistic regression analysis was carried out to examine the effects of each predictor on the level of service utilization. The degree of association was reported using the adjusted relative risk ratio (aRRR) with a corresponding 95% confidence interval, and statistical significance was declared at *p* < 0.05.

**Results:**

66.4% (95% CI: 64.9, 67.7) and 23.8% (95% CI: 23.3, 24.2) of women used maternal health services partially and adequately, respectively. Togo has the highest proportion of women getting adequate health care in the region, at 56.7%, while Nigeria has the lowest proportion, at 11%. Maternal education, residence, wealth index, parity, media exposure (to radio and television), enrolment in health insurance schemes, attitude towards wife beating, and autonomy in decision-making were identified as significant predictors of partial and adequate maternal health service uptake.

**Conclusion:**

The uptake of adequate maternal health services in the region was found to be low. Stakeholders should plan for and implement interventions that increase women's autonomy. Program planners and healthcare providers should give due emphasis to those women with no formal education and from low-income families. The government and the private sectors need to collaborate to improve media access and increase public enrolment in health insurance schemes.

## Contributions to the literature

- There is a notable lack of comprehensive studies in the West African region that delve into the spectrum of maternal health service utilization, which ranges from women who do not use these essential services to those who use them adequately.
- The current study aimed to assess the determinants of adequate and partial utilization of basic maternal health services at the individual and community levels by employing a relatively advanced model and the most recent nationally representative DHS data from 12 countries in the region.
- Finally, the findings of this study will help to formulate effective policies and strategies aimed at improving the existing dynamics of maternal health service usage, thereby boosting maternal health outcomes.

## Background

Despite a global decline in maternal mortality by roughly 44% from 1990 to 2015 [1], maternal health remains one of the top concerns for governments and policymakers across the globe, especially in low-resource settings [2]. It is a vital part of the United Nations' 2015 Sustainable Development Goals (SDGs), especially SDG target 3.1 aiming to reduce maternal mortality to less than 70 maternal deaths per 100,000 live births by 2030 [3, 4]. Despite this, maternal mortality (MMR) remains one of the primary causes of death among African women. The Sub-Saharan Africa (SSA) region, encompassing almost all West African countries, has relatively high maternal death rates, accounting for 70% of maternal deaths (202,000) of global maternal mortality [5]. The West African rates appear to be among the world’s highest. Approximately 31%, 36%, and 33% of maternal deaths occur during pregnancy, during delivery or in the early postpartum period, and from 1 week to 1 year following childbirth, respectively [6]. To prevent these terrible maternal deaths, the World Health Organisation (WHO) and other international agencies launched initiatives to provide high-quality maternal health services throughout pregnancy, childbirth, and postpartum periods [5, 7].

Maternal health refers to the health of women during pregnancy, childbirth, and the postnatal period to ensure that women and their newborns attain their maximum potential for health and well-being [8]. Adequate utilization of maternal health services namely Antenatal care (ANC), Skilled Delivery Service (SDS), and Postnatal Care (PNC) have been proven to enhance maternal health outcomes and have been identified as the remedy for lowering maternal mortality in developing countries [9, 10]. ANC plays an essential part in reducing mortality by identifying and managing complications early, ensuring optimal nutrition and prenatal education, and encouraging healthy behaviors [7]. Access to skilled delivery services is essential to ensure safe deliveries, manage potential childbirth-related complications, and respond promptly to emergencies [11, 12]. Postnatal care is critical for recognizing and treating complications that may arise following childbirth, such as postpartum hemorrhage and maternal and infant infections(sepsis) [13]. The delivery of those services adequately as a continuum has been promoted on an international level to enhance mother and child health, hence reducing morbidities and mortality [13]. It is also vital for reducing the disparities in maternal mortality between developed and developing nations and enabling women to lead productive and fulfilling lives [1, 4]. Receiving all three basic services (ANC, SDS, and PNC) in the form of a continuum of care (CoC) can prevent 71% of MMR, but only 37% if one of the services is missing from the continuum [14].

The growing socioeconomic inequality in maternal healthcare utilization is a major concern for low and middle-income countries (LMICs) like West African countries striving to meet the SDGs [15, 16]. According to current statistics, 66%, 80%, and 61% of women worldwide received ANC, SDS, and PNC services, respectively [11, 17, 18]. There are huge disparities between developed and developing countries. Per post-MDGs estimates, progress in service use remains substantially inconsistent between and within regions, with non-/underutilization of key maternal health services significantly higher among African countries, especially the Western ones [19, 20]. In Sub-Saharan Africa, the proportion of women who use health services remains below 70%, with 55%, 66%, and 53% receiving at least four ANC, SDS, and PNC, respectively [21, 22].

Low accessibility and affordability of services due to various technological, and economic constraints, and behavioral factors such as low health literacy and self-esteem, are the primary barriers to adequate maternal health service usage in resource-limited settings such as the West African region [23–25]. In addition, a lack of trained health workers, shortages of essential medical supplies, and poor accountability of health systems also prevent women from receiving or seeking care during pregnancy and childbirth [5, 26]. Furthermore, social elements such as access to education, ethnicity, and racial background, harmful gender stereotypes and/or inequalities, and external influences such as climate and humanitarian crises contribute to the low uptake of adequate maternal health services [27–30].

Historically, the Western African region has encountered maternal health challenges such as high maternal mortality rates and poor access to adequate maternal health services during pregnancy and postpartum period [1, 31]. However, there is a notable lack of comprehensive studies that delve into the spectrum of maternal health service utilization, which ranges from women who do not use these essential services to those who use them adequately. As a result, the current study sought to assess multilevel correlates of adequate and partial utilization of maternal health services (ANC, SDS, and PNC) in a single analytical framework using the nationally representative demographic and health survey (DHS) data of 12 West African countries. The current study aimed to fill the knowledge gap by thoroughly assessing the extent and determinants of maternal health service uptake, encompassing a wide spectrum from partial to full utilization. The findings of this study will help to formulate effective policies and strategies aimed at improving the existing dynamics of maternal health service usage, thereby boosting maternal health outcomes.

## Methods

### Data source, population, and study period

The current study was based on the most recent Demographic and Health Surveys (DHS) (2013–2021) of twelve West African countries. The data was obtained by appending each country's women's (IR) file, which provides information on maternal health services and all noteworthy variables. DHS is a nationally representative poll that is conducted in about 90 low- and middle-income countries every five years to collect data on key health indicators [32]. The current study included women who gave birth five years preceding the survey and had full details on the utilization of maternal health services. A total weighted sample of 89,504 women was considered as the analytical sample (Table 1).

**Table 1:** Description of the West African countries included in the analysis with their respective sample size, 2013–2021

Country	DHS Year	Total weighted sample size
Benin	2018	9,031(10.1)
Cote d'Ivoire	2014	5,212(5.8)
Ghana	2014	4,141(4.6)
Gambia	2020	5,367(6.1)
Guinea	2018	5,464(6.1)
Liberia	2020	4,026(4.5)
Mali	2018	6,605(7.4)
Mauritania	2020	7,695(8.6)
Nigeria	2018	21,801(21.3)
Niger	2013	8,002(8.9)
Sierra Leone	2019	7,312(8.2)
Togo	2014	4,848(5.4)
Total		89,504(100.0)

### Data collection tool and procedures

The data of each country were collected through face-to-face interviews by trained data collectors using structured questionnaires. Women were selected using a stratified, two-stage cluster sampling technique in which data were hierarchical (i.e., women were nested in households, and households were nested within clusters). Finally, this study considered appended DHS data of 12 West African countries, with a weighted sample of 89,504 women who gave birth within five years preceding the survey. The Demographic and Health Survey Sampling and Household Listing Manual developed by ICF International looks at the thorough sampling technique employed during DHS [33].

### Measurement of variables of the study

#### Outcome variable

The level of maternal health service utilization was the study's outcome variable, and it stemmed from three distinct types of care: ANC, SDS, and PNC. ANC uptake was defined as a binary outcome, with 0 denoting women who did not receive the service and 1 denoting those who received at least a visit during their most recent pregnancy. SDS was determined based on the place of birth, with 0 representing those who gave birth outside of a medical facility and 1 representing those who did so or who had assistance from a skilled healthcare provider during delivery (by a doctor, nurse, midwife, public health or certified community health worker). PNC receipt was dichotomized as 1, and 0, for women who had a postpartum visit within two months of giving birth, and for those who did not respectively. A composite index was created by combining all three variables to produce a single outcome variable called maternal health service utilization, which has a minimum and a maximum value of 0 and 3, respectively. The outcome variable was then further divided into three categories: not utilized (for those who used none of the three services); partially utilized (for those who used one or two of the services); and adequately utilized (for those who used all of the services). Previous studies employed similar classification approaches, as of ‘no’, ‘partial’, and ‘adequate’ service uptake [34, 35].

#### Explanatory variables

Potential factors of maternal health service use have been extracted from the data set after reviewing related and recent literature. Because the DHS data is hierarchical, the variables were categorized as individual- and community-level factors. Individual-level factors were features that were unique to each woman and were classified as sociodemographic, obstetric, and health-care-related characteristics. Community-level factors, on the other hand, were attributes shared by all women living in the same community (cluster), such as place of residence and community poverty (Table 2).

**Table 2:** Individual and community-level factors that are supposed to affect the uptake of maternal health services in West African countries, 2013–2021

Variables	Description	Category
Individual-level factors		
Age	The respondent's age, expressed in years, at the time of the survey	1. 15–19,
		2. 20–34
		3. 35–49^a^
Marital status	Percentage of women according to the current status of marriage or cohabitation	1. Married
		2. Never married
		3. Others^a^
Women educational attainment	Percent distribution of women ages 15–49 by the highest level of schooling attended or completed	1. No education^a^,
		2. Primary
		3. Secondary
		4. Higher
Occupational status	Percent distribution of women and men employed in the 12 months preceding the survey by occupation	1. Unemployed^a^
		2. Employed
Family size	Number of household members at the time of data collection	1. ≤ 5^a^
		2. > 5
Wealth index	Calculated using easy-to-collect data on a household's ownership of selected assets, such as televisions and bicycles; materials used for housing construction; and types of water access and sanitation facilities	1. Richest^a^
		2. Richer
		3. Middle
		4. Poorer
		5. Poorest
Sex of head of Household	Percent distribution of households by sex of head of household	1. Female
		2. Male^a^
Parity	The number of living children the woman had at the time of the survey	1. Nulliparous^a^
		2. Primiparous
		3. Multiparous
		4. Grand multiparous
Total children ever born	Percentage of women with a specified number of children ever born at the time of the survey	1. No^a^
		2. One
		3. Two to four
		4. Five and more
Pregnancy status during last childbirth	Percent distribution of births to women aged 15–49 in the 5 years preceding the survey, including current pregnancies, by planning status of the birth – (*i*) wanted then, (*ii*) wanted later, or *(iii*) not wanted at all	1. Unwanted (*ii& iii)*
		2. Wanted (*i*)^a^
Difficulty in accessing healthcare	Percentage of women age 15–49 who reported that they have serious problems in accessing health care for themselves when they are sick, by type of problem: 1. Getting permission to go for treatment 2. Getting money for treatment 3. Distance to the health facility and the responses were categorized as ‘Not big problem’ or ‘Big problem’	1. Not big problem
		2. Big problem^a^
Covered by health insurance	Percentage of women and men ages 15–49 covered by any health insurance schemes	1. Yes
		2. No^a^
Media exposure	Number of women age 15–49 who are exposed to specific media with various frequencies: read a newspaper, watch television, and listen to radio	1. Not at all^a^
		2. Less than once a week
		3. ≥ Once a week
Autonomy in decision-making ^**a**^	The aggregate distribution of currently married women aged 15–49 who usually makes decisions about their own health care, large household purchases, and visits to family or relatives	1. Low^a^
		2. Medium
		3. high
The overall attitude towards the wife beating ^**b**^	A proportion of women aged 15 to 49 agreed or disagreed that a husband is justified in hitting or beating his wife for at least one of the following reasons: burning food, arguing with him, going out without notifying him, neglecting the children, refusing to have sexual intercourse with him	1. Low
		2. Moderate
		3. High^a^
**Community-level factors**		
Residence	The area where respondents lived when the survey was conducted	1. Urban
		2. rural^a^
Community level poverty	Described as the proportion of respondents who lived in the cluster's worst housing conditions. The cluster's overall poverty can be estimated and classified as low, moderate, or high by adding together the individual households with the lowest wealth indices	1. Low^a^
		2. Moderate
		3. High

^a^Reference category

^a^*Autonomy in decision-making*: Responses to questions about who takes final decisions for the family on big home purchases, visits to family, and health care were used to assess decision-making autonomy. (i) respondent alone, (ii) joint decision (respondent and husband/partner), (iii) husband/partner alone, (iv) someone else, and (v) others were the response categories. For each question, responses (i) or (ii) were assigned a value of 1, indicating high decision-making capacity, while the remaining responses were assigned a value of 0, indicating low power. The responses on all three dimensions were added together to yield an overall score that goes from 0 to 3. Finally, a composite score was then divided into three distinct groups: low, middle, and high for scores "0 to 1", "2", and "3", respectively [36, 37].

^b^*Acceptance of wife beating*: Was evaluated using five criteria: (i) Beating justified if she ignores children, (ii) Beating rationalized if she argues with her husband, (iii) Beating justified if she refuses to have sex, (iv) Beating justified if she leaves the house without her husband's permission, and (v) Beating justified if she burns foods. The response alternatives for each item were (i) no, (ii) yes, and (iii) don't know. Response (i) was given a 0 to indicate that it wasn't accepted, while the other responses were given a 1 to indicate that they agreed the wife beating. The responses to those five questions were totalled to get a composite score ranging from 0 to 5. A lower score on this indicator is seen as indicating a larger sense of entitlement and self-esteem, as well as women's higher position. Finally, for scores "0 to 2", "3 to 4", and "5", a composite score was separated into three distinct groups: low, moderate, and high respectively [38, 39].

### Statistical analysis and data management

The data was recoded, cleaned, and analyzed using STATA software version 16. Initially, the most recent data sets of the 12 countries were appended together to generate a single data pool for Western African countries. The data were weighted by applying a weighting factor $(\frac{\mathbf{v}005}{1000000})$ to minimize under- or over-representation of the data in the surveys due to differential selection among strata. Using the *svyset* command, the data was further structured as survey data. Descriptive statistics such as frequency, mean, and percentage were used to describe the respondents' background characteristics. Chi-square tests were carried out to look into the distribution of individual and community-level factors across various service utilization categories. A Variance Inflation Factor (VIF) has been used to test for multicollinearity among variables, and there was none (the VIF ranged from 1.02 to 3.97, with a mean of 1.65).

### Multilevel multinomial regression

Multilevel modeling was used to deal with the hierarchical nature of the DHS data, where women were nested within households and households were nested within clusters. Using a multilevel analysis for such data allows us to prevent biased parameter estimations that may emerge from a single-level analysis [40]. A multilevel mixed-effect multinomial logistic regression was a good fit for the current study as the outcome variable had more than two categories (not utilized, somewhat utilized, and completely utilized).

### Modeling building approaches

#### Fixed effect model

After running a bivariable multinomial regression, variables with *p*-values < 0.25 were imported into a multilevel multivariable multinomial logistic regression. Then, multilevel multinomial logistic regression models had been used to determine significant predictors of service uptake. In the analysis, the base outcome is ‘no’ (women who did not obtain any of the three services). To estimate the model, a generalized structural equation modeling (GSEM) (with a logit link function) was carried out by using the "*gsem*" STATA command. The estimated logit coefficients were exponentiated to yield adjusted relative risk ratios (aRRRs) for ease of interpretation. Finally, aRRR was reported, along with its corresponding 95% confidence interval (CI). Variables with a p-value < 0.05 were considered significant predictors of levels of partial- and full-service uptake.

#### Random effect models

Using a hierarchical approach, four different nested random intercept models (Models 1, 2, 3, and 4) were fitted. Model one (null model) has only the intercept and no explanatory variables. The second and third models, respectively, incorporate only individual and community-level characteristics. The fourth (final) model was fitted using both model 2 (individual-level) and model 3 (community-level) variables. To quantify the random effects (variability in service uptake between clusters), the intraclass correlation coefficient (ICC) along with the proportional change in variance (PCV) were computed.

$ICC=\frac{{\text{var}}\left({\text{b}}\right)}{{\text{Var}}\left({\text{b}}\right)+{\text{Var}}({\text{w}})}$ Where Var(b) is the group variance and Var(w) is a predicted individual variance component, which equals π2/3 ≈3.29. Proportional Change in Variance (PCV) was estimated as

$PCV=\frac{({\text{Va}}-{\text{Vb}})}{{\text{Va}}}*100$, where, V_a_ is the variance of the initial model (null model), and V_b_ = variance of the subsequent models (models 2, 3, and 4).

The best model fit was selected by using deviance = -2 * (Log Likelihood (LL), Schwarz's Bayesian Information Criterion (BIC), and Akaike's information criterion (AIC) to interpret the results. Finally, the fourth model with the lowest deviance, AIC, and BIC values was chosen as the best-fit model for the current study (Table 5).

## Results

### Maternal health services uptake by characteristics of respondents

The analyses of the current study have relied on a total weighted sample of 89,504 women from 12 West African nations with the most recent DHS data. Nigeria and Liberia had the highest (21.3%) and lowest (4.5%) proportions of study participants, respectively. The mean (± SD) age of study participants was 29.50(± 7.32), with nearly half (46.6%) being in the age bracket of 25–34 years. The vast majority (90.2%) were married, and more than half (53.7%) had no formal education. Rural residents made up roughly two-thirds of the population (61.5%). Almost half of the respondents (51.0%) were multiparous (had two to four living children). The majority of women, 71,758 (80.2%), wanted their last pregnancy. A considerable majority of women (49.8%) had low autonomy in decision-making (Table 3).

**Table 3:** Frequency distribution and reported prevalence of maternal health services utilization across different characteristics of women in West Africa region, 2012–2021

Variable categories	Total weighted sample size [N(%)]	Overall maternal health service utilization			
		Not utilized [n(%)]	Partially utilized [n(%)]	Adequately utilized [n(%)]	Test statistics
	*N* = 89,504	8,740( 9.8)	59,407(66.4)	21,357(23.9)	
**Age in years**					
15–24	24,067(26.9)	2,319( 9.6)	16,198(67.3)	5,550(23.1)	X^2^ = 43.04 *p* = 0.031
25–34	41,732(46.6)	3,828(9.2)	27,693(66.4)	10,211(24.4)	
35–49	23,706(26.5)	2,593(10.9)	15,516(65.5)	5,596(23.6)	
**Marital status**					
In marital relation	80,739(90.2)	8,313(10.3)	53,514(66.3)	18,912(23.4)	X^2^ = 238.28
Not in union	8,765(9.8)	428(4.9)	5,893(67.2)	2,445(27.9)	*p* < 0.001
**Educational status**					
No education	48,035(53.7)	7,459(15.5)	31,656(65.9)	8,921(18.6)	X^2^ = 1428.18 *p* < 0.001
Primary	17,278(19.3)	849(4.9)	11,767(68.1)	4,662(27.0)	
Secondary/ higher	24,190(27.0)	432(1.8)	15,984(66.1)	7,774(32.1)	
**Occupational status**					
Employed	64,967(72.6)	6,726(10.4)	42,393(65.2)	15,848(24.4)	X^2^ = 63.71
Un employed	24,537(27.4)	2,014(8.3)	17,014(70.3)	5,509(22.8)	*p* < 0.001
**Residence**					
Urban	34,217(38.2)	1,059(3.1)	22,613(66.1)	10,546(30.8)	X^2^ = 3208.28
Rural	55,287(61.8)	7,681(13.9)	36,794(66.6)	10,811(19.5)	*p* < 0.001
**Family size**					
≤ 5memeber	34,430(38.5)	2,856( 8.3)	22,769(66.1)	8,805(25.6)	X^2^ = 92.37
> 5 member	55,074(61.5)	5,884(10.7)	36,638(66.5)	12,552(22.8)	*p* < 0.001
**Head of household**					
Male	73,498(82.1)	7,787(10.6)	48,546(66.0)	17,165(23.4)	X^2^ = 343.31
Female	16,006(17.9)	953(5.9)	10,861(67.9)	4,192(26.2)	*p* < 0.001
**Wealth index combined**					
Poorest	19,001(21.2)	3,864(20.3)	12,166(64.0)	2,971(15.7)	X^2^ = 1921.37 *p* < 0.001
Poorer	18,861(21.0)	2,725(14.4)	12,558(66.6)	3,578(19.0)	
Middle	18,111(20.2)	1,380(7.6)	12,468(68.9)	4,263(23.5)	
Richer	17,619(19.7)	625(3.6)	12,041(68.3)	4,953(28.1)	
Richest	15,912(17.8)	146(0.9)	10,175(63.9)	5,591(35.1)	
Community Poverty					
Low	32,321( 36.1)	2,391(7.4)	22,401(69.3)	7,529(23.3)	X^2^ = 121.30 *p* < 0.001
Middle	29,294(32.7)	2,458(8.4)	19,236(65.7)	7,601(26.0)	
High	27,889(31.2)	3,892(14.0)	17,770(63.7)	6,227(22.3)	
**Parity**					
Nulliparous	1,029(1.2)	143(13.9)	714(69.4)	172(16.7)	X^2^ = 832.21 *p* < 0.001
Primiparous	18,591(20.8)	1,202(6.5)	12,057(64.9)	5,332(28.7)	
Multiparous	45,612(51.0)	4,174(9.2)	30,330(66.5)	11,108(24.3)	
Grand multiparous	24,272(27.1)	3,221(13.3)	16,305(67.2)	4,746(19.5)	
**Children ever born**					
One	17,177(19.2)	1,028(6.0)	11,122(64.8)	5,027(29.2)	X^2^ = 921.27 p < 0.001
2–4	41,851(46.8)	3,636( 8.7)	27,734(66.3)	10,481(25.0)	
≥ 5	30,477(34.0)	4,077(13.4)	20,551(67.4)	5,849 (19.19)	
**Planning status of last pregnancy**					
Wanted	71,758(80.2)	7,642(6.2)	47,412(67.6)	16,704(26.2)	X^2^ = 293.22 *p* < 0.001
Unwanted	17,746(19.8)	1,098 (10.7)	11,995(66.1)	4,653(23.3)	
**Reading newspaper**					
Not at all	81,630(91.3)	8,612(10.5)	54,493(66.8)	18,525(22.7)	X^2^ = 952.77 *p* < 0.001
< once a week	5,303(5.9)	98( 1.8)	3,423(64.6)	1,782(33.6)	
At least once a week	2,539(2.8)	27(1.1)	1,479(58.3)	1,032(40.6)	
**Listening to a radio**					
Not at all	38,561(43.1)	5,678(14.8)	26,041(67.5)	6,842(17.7)	X^2^ = 2213.16 *p* < 0.001
≥ once a week	22,809(25.5)	1,639(7.2)	15,320(67.2)	5,850 (25.6)	
Once and more a week	28,134(31.4)	1,423(5.1)	18,046(64.1)	8,665(30.8)	
**Watching TV**					
Not at all	50,133(56.0)	7,405( 14.8)	33,713(67.2)	9,016(18.0)	X^2^ = 1192.42 *p* < 0.001
< once a week	15,398(17.2)	794( 5.2)	10,523(68.3)	4,081(26.5)	
≥ once a week	23,951(26.8)	541(2.3)	15,159(63.3)	8,251(34.4)	
**Covered by health insurance**					
Yes	5,267(5.9)	72(1.4)	2,636(50.0)	2,559(48.6)	X^2^ = 1091.23
No	84,237(94.1)	8,668(10.3)	56,771(67.4)	18,798(22.3)	*p* < 0.001
**Distance to a health facility**					
Big problem	31,674(35.4)	4,572(14.4)	20,847(65.8)	6,254(19.78)	X^2^ = 391.12
Not a big problem	57,830(64.6)	4,168(7.2)	38,560(66.7)	15,103(26.1)	*p* < 0.001
**Getting money**					
Big problem	47,675(53.3)	5,784(12.1)	31,353(65.8)	10,538(22.1)	X^2^ = 647.60
Not a big problem	41,829(46.7)	2,956(7.1)	28,054(67.1)	10,819(25.8)	*p* < 0.001
**Obtaining permission**					
Big problem	17,054(19.0)	2,649(15.5)	11,299(66.3)	3,106(18.2)	X^2^ = 1091.23
Not a big problem	72,450(81.0)	6,091(8.4)	48,108(66.4)	18,251(25.2)	*p* < 0.001
**Attitude toward wife beating**					
Low	62,034(69.3)	5,160( 8.3)	41,125(66.3)	15,749(25.4)	X^2^ = 847.31 *p* < 0.001
Middle	16,058(17.9)	1,584(9.9)	10,716(66.7)	3,758(23.4)	
High	11,413(12.8)	1,996(17.5)	7,566(66.3)	1,850 (16.2)	
**Autonomy in decision-making**					
Low	44,592(49.8)	6,193(13.9)	29,235(65.6)	9,163(20.5)	X^2^ = 1007.33 *p* < 0.001
Middle	11,164(12.5)	787(7.1)	7,261(65.0)	3,116(27.9)	
High	33,748(37.7)	1,760(5.2)	22,911(67.9)	9,077(26.9)	

There were significant disparities across the sociodemographic, socioeconomic, and obstetric characteristics of the respondents. Women aged 25–34 years and 15–24 years had the highest rates of partial and adequate utilization of health services, at 66.4% and 26.0%, respectively. In terms of education, partial and adequate uptake of maternal health services was greater among women who attended primary, and higher education, at 68.2% and 32.1%, respectively. Non-use of health services, on the other hand, was higher (15.5%) among women with no formal education compared to women with higher education (1.8%). Partial utilization of health services was higher (66.6%) among women living in rural areas, whereas adequate utilization was higher (30.8%) among women living in urban areas. The richest and middle wealth index categories had the highest proportions of women receiving adequate and partial maternal health services, at 35.1% and 68.9%, respectively. Regarding obstetric characteristics, primiparous women (28.7%) and women with wanted pregnancies (26.2%) received the highest proportion of adequate maternal health services. Women enrolled in health insurance schemes reported the highest proportion (48.6%) of adequate maternal health service utilization. Women who were exposed to media one or more times per week had the highest proportion of adequate usage of health services (34.4% for television and 30.8% for radio) (Table 3).

### Overall maternal health service utilization

**Fig. 1:**
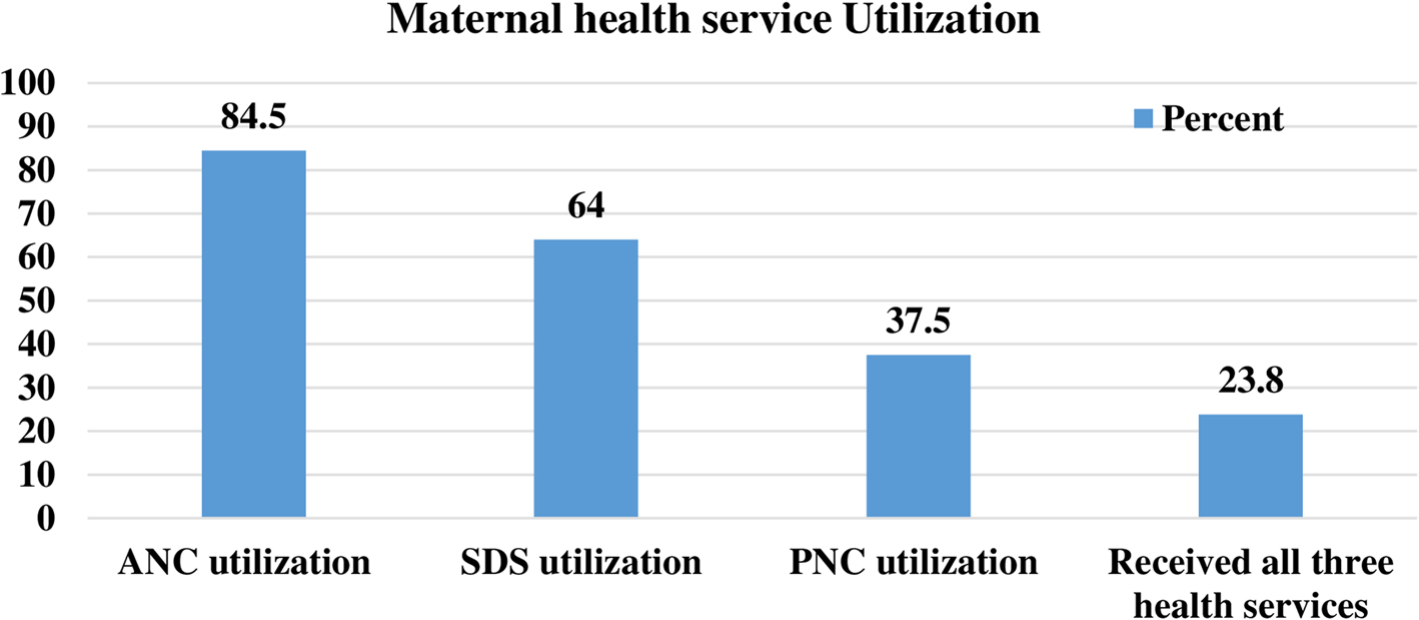
The uptake of key maternal health services in the West African region, 2013–2021

In West African countries, 84.5%, 64.0%, and 37.5% of women received at least one ANC visit, SDS, and PNC, respectively (Fig. 1). The highest and lowest prevalence of receiving ANC have been recorded in Gambia and Nigeria, with 98.8% and 73.9%, respectively. Benin and Niger exhibited the highest proportions of SDS and PNC, making up 86.8% and 61.9%, respectively.

Regarding the overall use of maternal health services, 66.4% (95% CI: 64.9, 67.7) and 23.8% (95% CI: 23.3, 24.2) of women, respectively, used partially and adequately. Togo, with 56.7%, and Nigeria, with 11%, represent countries with the highest and lowest proportions of women receiving adequate health services in the region, respectively. Nigeria, and Sierra Leone on the other hand, had the highest and lowest proportions of women not receiving any maternal health services, at 67.8% and 0.6%, respectively (Table 4).

**Table 4:** The distribution of maternal health service utilization across West African countries, 2013–2021

Country	Total weighted sample size (*N* = 89,504)	Utilization of each service			Overall health service utilization		
		ANC	SDS	PNC	Not utilized	Partially utilized	Adequately utilized
		[n (%)]	[n (%)]	[n(%)]	[n (%)]	[n (%)]	[n(%)]
Benin	9,031(10.1)	7,814(86.5)	7,842(86.8)	1,728(19.1)	601(6.7)	6,990(77.4)	1,440(15.9)
Cote d'Ivoire	5,212(5.8)	4,788(91.9)	3,185(61.1)	3,659(70.2)	182(3.5)	2,768(53.1)	2,263(43.4)
Ghana	4,141(4.6)	4,013(96.9)	3,130(75.6)	2,996(72.3)	51(1.2)	1,802(43.5)	2,288(55.3)
Gambia	5,367(6.0)	5,306(98.8)	4,644(86.5)	2,913(54.3)	8(0.2)	3,041(56.7)	2,319(43.2)
Guinea	5,464(6.1)	4,548(83.2)	2,957(54.1)	1,923(35.2)	636(11.6)	3,754(68.7)	1,074(19.7)
Liberia	4,026(4.5)	3,884(96.5)	3,321(82.4)	991(24.6)	34(0.9)	3,386(84.1)	605(15.0)
Mali	6,605(7.4)	5,174(78.3)	4,647(70.4)	1,679(25.4)	746(11.3)	4,762(72.1)	1,096(16.6)
Mauritania	7,695(8.6)	6,330(82.3)	5,662(73.6)	1,427(18.6)	665(8.6)	6,155(80.0)	875(11.4)
Nigeria	21,801(21.3)	16,114(73.9)	9,327(42.8)	4,550(20.9)	4,642(67.8)	14,761(11.0)	2,398(11.2)
Niger	8,002(8.9)	6,817(85.2)	2,717(33.9)	4,889(61.9)	931(11.6)	4,995(62.4)	2,075(25.9)
Sierra Leone	7,312(8.2)	6,327(86.5)	6,194(84.7)	3,329(45.5)	45(0.6)	5,093(69.7)	2,174(29.7)
Togo	4,848(5.4)	4,482(92.5)	3,644(75.2)	3,487(71.9)	199(4.1)	1,899(39.2)	2,750(56.7)

### Random effect (measures of variation)

The intraclass correlation coefficient (ICC) and Proportional Change in Variance (PCV) statistics were calculated for the measures of variation (random effects). According to the results of the null model (model 1), between-cluster variations account for 15.6% of the total variance in maternal health service use (ICC = 0.156, *p* < 0.001). The between-cluster variation decreased from 15.6% to 6% as we go from a null model to full mode that contains all individual- and community-level variables. This suggests that the variances across the clusters can explain the differences in the probability of receiving using health services partially and adequately. Factors at the individual and communal levels collectively explained 48.7% of the variation observed in the null model (PCV = 48.8%). The values of AIC, BIC, and Deviance decreased continually as we moved from model 1 (the empty model) to model 4 (the full model), indicating that the final model built throughout the study had adequate goodness of fit. Following the comparison, the fourth model with the lowest deviance (136,878.4) was selected as the best-fit model and used for the interpretation of the findings (Table 5).

**Table 5:** Random intercept and model fit statistics comparison of multilevel mixed effect multinomial logistic regression model

**Measures**	Model 1 (null model)	Model 2 (individual-level)	Model-3 ( community-level)	Model 4 (full model)
**Random effects**				
Variance	0.61	0.43	0.32	0.21
ICC	15.6%	11.6%	8.9%	6.00%
PCV	Reference	24.4%	29.3%	48.8%
**Model fitness**				
AIC	148,256.9	137,392.5	14,602.74	137,020.4
BIC	148,285.1	138,003.9	14,781.7	137,688.3
Log-likelihood	-74,125.4	-68,631.2	-72,422.3	-68,439.2
Deviance	148,250.8	137,262.4	144,844.6	136,878.4

### Fixed effect results: predictors of maternal health service utilization

A bivariable multinomial regression was carried out, and the majority of variables were associated with maternal health service utilization. Individual- and community-level attributes that showed an association were inserted into a multivariable multinomial logistic regression (Table 6).

**Table 6:** Results of bivariable multinomial logistic regression analysis to identify predictors of partial and adequate maternal health service uptake in West African countries, 2013–2021

Variable categories	Partially utilized cRRR (95% CI)	Adequately utilized cRRR (95% CI)
**Individual-level factors**		
**Age in years**		
15–24	1.17(1.09, 1.25)	1.11(1.03, 1.19)
25–34	1.21(1.13, 1.28)	1.24(1.15, 1.32)
35–49	Ref	Ref
**Marital status**		
In marital relationship	Ref	Ref
Not in union	1.14(0.90, 1.40)	1.51(1.22, 1.83)
**Educational status**		
No education	Ref	Ref
Primary	3.26( 3.00, 3.55)	4.59(4.19, 5.02)
Secondary and above	4.70(2.81, 5.70)	6.02(4.44, 7.79)
**Occupational status**		
Employed	0.74(0.70, 0.79)	0.86(0.80, 0.92)
Un employed	Ref	Ref
**Family size**		
≤ 5memeber	1.25(1.21, 1.35)	1.44(1.36, 1.53)
> 5 member	1	1
**Head of household**		
Male	Ref	Ref
Female	1.63(1.38, 1.78)	2.00( 1.82, 2.18)
**Wealth index combined**		
Poorest	Ref	Ref
Poorer	1.46(1.37, 1.55)	1.70(1.58, 1.84)
Middle	1.87(1.65, 2.10)	4.01(3.67, 4.39)
Richer	3.12(2.52, 4.78)	5.31( 3.22, 6.51)
Richest	8.12(6.40, 10.69)	12.88(10.26, 15.31)
**Parity**		
Nulliparous	Ref	Ref
Primiparous	2.00(1.60, 2.51)	3.70(2.81, 4.86)
Multiparous	1.45(1.17, 1.80)	2.22(1.70, 2.89)
Grand multiparous	1.01(0.81, 1.26)	1.23(0.94, 1.60)
**Planning status of last pregnancy**		
Wanted	1.76(1.63, 1.89)	1.94( 1.79, 2.10)
Unwanted	Ref	Ref
**Reading newspaper**		
Not at all	Ref	Ref
Less than once a week	1.54(1.33, 4.10)	1.49(1.11, 1.91)
At least once a week	2.51(1.84, 2.97)	1.34(1.12, 1.46)
**Listening to a radio**		
Not at all	Ref	Ref
Less than once a week	2.04(1.90, 2.18)	2.96(2.74, 3.19)
≥ Once per week	2.76(2.57, 2.97)	5.05(4.67, 5.45)
**Watching TV**		
Not at all	Ref	Ref
Less than once a week	2.91(2.66, 3.18)	3.22(2.83, 3.64)
≥ Once per week	3.41(2.73, 4.83)	4.51(3.23, 5.35)
**Distance to a health facility**		
Big problem	Ref	Ref
Not a big problem	2.02(1.92, 2.13)	2.64(2.49, 2.81)
**Getting money**		
Big problem	Ref	Ref
Not a big problem	1.75(1.65, 1.84)	2.00(1.89, 2.13)
**Obtaining permission**		
Big problem	Ref	Ref
Not a big problem	1.85(1.74, 1.96)	2.55(2.38, 2.73)
**Covered by health insurance**		
Yes	5.55( 4.25, 7.25)	16.28(12.46, 21.28)
No	Ref	Ref
**Attitude toward wife beating**		
Low	2.10(1.96, 2.24)	3.29(3.03, 3.56)
Middle	1.78(1.64, 1.93)	2.55( 2.31, 2.82)
High	Ref	Ref
**Autonomy in decision-making**		
Low	Ref	Ref
Middle	1.95(1.78, 2.13)	2.67(2.43, 2.94)
High	2.76(2.59, 2.93)	3.48(3.25, 3.73)
**Community-level factors**		
**Residence**		
Urban	Ref	Ref
Rural	0.22(0.20, 0.24)	0.14( 0.12, 0.18)
**Community poverty**		
Low	2.05(1.92, 2.19)	1.97(1.83, 2.11)
Middle	1.71(1.61, 1.82)	1.93(1.80, 2.06)
High	Ref	Ref

Key: *Ref* Reference category, *cRRR* crude relative risk ratio

### Multilevel multivariable multinomial regression analysis

To identify significant predictors of partial and adequate utilization of maternal health services, a multilevel multivariable multinomial regression analysis was carried out. Maternal education, residence, wealth index, parity, media exposure, distance to access health care, enrolment in health insurance schemes, attitude towards wife beating, and autonomy in decision-making were identified as significant predictors of partial and adequate health service uptake.

The likelihood of accessing health services partially and adequately was 2.94 [aRRR = 2.94; 95% CI: 2.63, 3.29] and 3.22 [aRRR = 3.22; 95% CI: 2.85, 3.63] times higher among women with secondary and higher education, respectively, as compared to women with no formal education. Women in the richest wealth quintile, respectively had 4.89 [aRRR = 4.89; 95% CI: 4.08, 5.85] and 6.57 [aRRR = 6.57; 95% CI: 5.46, 7.92] times higher chance of receiving partial and adequate maternal health services as compared to their counterparts in the poorest quintile. As compared to Grand multiparous, primiparous women had an increased likelihood of receiving health services partially and adequately by 1.53[aRRR = 1.53; 95% CI: 1.22, 1.93] and 3.21[aRRR = 3.21; 95% CI: 2.36, 4.32] times, respectively.

Regarding media exposure, women who listen to the radio one or more times per week have higher odds of partially and adequately utilizing health services than women who don't listen to radio at all—by a factor of 1.55 [aRRR = 1.55; 95% CI: 1.44, 1.66] and 2.34 [aRRR = 2.34; 95% CI: 2.15, 2.54] respectively. Similarly, women who watched television one or more times per week had a 2.16 times greater chance of receiving adequate health care than women who did not watch TV at all [aRRR = 2.16; 95% CI: 1.91, 2.44]. Women who were covered by health insurance schemes had 3.78 [aRRR = 3.78; 95%CI: 2.92, 4.88] and 7.91 [aRRR = 7.91; 95%CI: 6.17, 10.35] times a higher likelihood of receiving health care partially and adequately. Attitude toward wife beating was also identified as a significant predictor of service usage. Women with low acceptance of wife beating had a 1.63 [aRRR = 1.63; 95%CI: 1.49, 1.78] times higher risk of receiving health services adequately. Finally, participants with higher decision-making autonomy, respectively had 1.92 [aRRR = 1.92; 95% CI: 1.59, 2.25] and 2.67 [aRRR = 2.67; 95% CI: 2.43, 2.94] times higher chance of receiving health care partially and adequately than those with lower decision-making autonomy. As compared to women who resided in urban areas, women who lived in rural areas had a 29% [aRRR = 0.71; 95% CI: 0.65, 0.78] and 41% [aRRR = 0.59; 95% CI: 0.54, 0.65] lower chance of receiving health services partially and adequately, respectively (Table 7).

**Table 7:** Results of multilevel multivariable multinomial logistic regression analysis to identify predictors of partial and adequate maternal health services uptake of in West African countries, 2013–2021

Variable categories	Model II (individual-level factors)		Model-III (community-level) factors		Model-IV (Full model)	
	Partially utilized aRRR (95%CI)	Fully utilized aRRR (95% CI)	Partially utilized aRRR (95%CI)	Fully utilized aRRR (95% CI)	Partially utilized aRRR (95%CI)	Fully utilized aRRR (95% CI)
**Age in years**						
15–24	1.17(1.09, 1.25)	1.17(0.96, 1.74)			0.85(0.78, 0.93)	0.86(0.79, 1.24)
25–34	1.21(1.13, 1.28)	0.98(0.97, 1.47)			0.97(0.91, 1.04)	0.97(0.83, 1.15)
35–49	Ref	Ref			Ref	Ref
**Marital status**						
Not in union	0.74(.64,0.85)	0.83(0.74, 1.24)			0.91(0.78, 1.14)	1.42(0.93, 1.65)
In marital relationship	Ref	Ref			Ref	Ref
**Educational status**						
Secondary/higher	2.96(2.64, 3.31)**	3.05(2.70, 3.44)**			2.94(2.63, 3.29)**	3.22(2.85, 3.63)**
Primary	2.16(1.98, 2.36)**	2.56(2.32, 2.82)**			2.14(1.96, 2.31)**	2.56(2.32, 2.82)**
No education	Ref	Ref			Ref	Ref
**Occupation**						
Employed	0.72(0.67, 1.16)	0.87(0.83. 1.19)			0.98(0.93, 1.04)	0.81(0.72, 1.03)
Un employed	Ref	Ref			Ref	Ref
**Family size**						
≤ 5memeber	0.98(0.92, 1.04)	0.94(0.87, 1.14)			0.96(0.91, 1.02)	0.96(0.89, 1.03)
> 5 member	Ref	Ref			Ref	Ref
**Household’s Head**						
Female	1.28(1.16, 1.40)**	1.17(0.88, 1.39)			1.33(0.98, 1.45)	1.29(0.98, 1.42)
Male	Ref	Ref			Ref	Ref
**Wealth index**						
Richest	7.07(5.79, 8.62)**	10.1(8.2,12.39)**			4.89(4.08, 5.85)**	6.57(5.46, 7.92)**
Richer	3.08(2.75, 3.43)**	4.02(3.56, 4.54)**			2.45(2.20, 2.72)**	2.90(2.59, 3.25)**
Middle	1.93(1.78, 2.09)*	2.36(2.15, 2.59)**			1.74(1.61, 1.88)**	2.04(1.87, 2.23)**
Poorer	1.22(1.14, 1.30)*	1.36(1.25, 1.47)*			1.25(1.17, 1.33)*	1.31(0.91, 1.42)
Poorest	Ref	Ref			Ref	Ref
**Parity**						
Grand multiparous	1.25(0.97, 1.61)	1.41(0.98, 1.92)			1.05(0.83, 1.32)	1.41(0.98, 1.92)
Multiparous	1.45(1.17, 1.80)	1.98(1.47, 2.68)*			1.16(0.93, 1.46)	2.04(1.52, 2.75)**
Primiparous	1.78(1.39, 2.28)**	3.13(2.31, 4.23)**			1.53(1.22, 1.93)*	3.21(2.36, 4.32)**
Nulliparous	Ref	Ref			Ref	Ref
**Pregnancy status**						
Wanted	1.41(1.28, 1.55)*	1.50(1.36, 1.67)*			1.29(1.18, 1.41)*	1.28(0.98, 1.40)
Unwanted	Ref	Ref			Ref	Ref
**Reading newspaper**						
At least once a week	1.01(0.78, 1.31)	1.14(0.78, 1.66)			0.89(0.64, 1.24)	1.20(0.83, 1.75)
< once a week	0.97(0.67, 1.40)	1.03(0.79, 1.35)			1.13(0.90, 1.41)	1.04(0.79, 1.35)
Not at all	Ref	Ref			Ref	Ref
**Listening to a radio**						
≥ Once per week	1.60(1.49, 1.73)	2.24 (2.07,2.44)**			1.55(1.44, 1.66)**	2.34(2.15, 2.54)**
< once a week	1.31(1.22, 1.41)*	1.59(1.46, 1.73)*			1.36(1.27, 1.46)*	1.64(1.51, 1.78)*
Not at all	Ref	Ref			Ref	Ref
**Watching TV**						
≥ Once per week	1.64(1.46, 1.84)**	2.64(1.76, 3.03)**			1.48(1.33, 1.64)	2.16(1.91, 2.44)**
< once a week	1.36(1.24, 1.50)*	1.31(1.12, 1.46)*			1.26(0.96, 1.38)	1.51(1.36, 1.68)*
Not at all	Ref	Ref			Ref	Ref
**Distance to a health facility**						
Not a big problem	1.23(1.15, 1.31)*	1.31(0.94, 1.49)			1.39(1.23, 0.39)	1.51(1.30, 1.79)**
Big problem	Ref	Ref			Ref	Ref
**Getting money**						
Not a big problem	1.04(0.97, 1.11)	0.91(.83, 1.07)			1.10(0.93, 1.17)	0.94(0.86, 1.11)
Big problem	Ref	Ref			Ref	Ref
**Obtaining permission**						
Not a big problem	1.37(1.28, 1.47)*	1.72(1.58, 1.86)*			1.13(0.95, 1.42)	1.64(1.53, 1.77)*
Big problem	Ref	Ref			Ref	Ref
**Using Health insurance**						
Yes	2.42(1.85, 3.17)**	5.55(4.22, 7.29)**			3.78(2.92, 4.88)**	7.91(6.17, 9.98)**
No	Ref	Ref			Ref	Ref
**Attitude on wife beating**						
Low	1.54(1.41, 1.68)**	2.02(1.82, 2.23)**			1.19(0.93, 1.36)	1.08(0.93, 1.25)
Middle	1.34(1.25, 1.44)*	1.65(1.52, 1.80)*			1.34(0.96, 1.62)	1.63(1.49, 1.78)**
High	Ref	Ref			Ref	Ref
**Decision-making autonomy**						
High	1.76(1.61, 1.90)**	2.01(1.85, 2.18)**			1.92(1.59, 2.25)^**^	2.67(2.43, 2.94)
Middle	1.42(1.37, 1.89)*	1.64(1.48, 1.81)*			1.72(1.60, 1.84)*	1.36(1.14, 1.93)*
Low	Ref	Ref			Ref	Ref
**Residence**						
Rural			0.25(0.23, 0.27)**	0.14(0.13, 0.16)**	0.71(0.65, 0.78)	0.59(.54, 0.65)**
Urban			Ref	Ref	Ref	Ref
**Community poverty**						
Low			2.67(2.22, 3.22)	2,24(1.86, 2.70)**	1.41(1.34, 1.96)*	1.67(1.33, 2.06
Middle			2.06(1.67, 2.56)	2.15(1.73, 2.68)**	1.14(0.95, 1.38)	0.87(0.72, 1.05)
High			Ref	Ref	Ref	Ref

Key: *Ref*. Reference category, *aRRR* adjusted relative risk ratio ^*^significant at *p*-value < 0.005, ^**^ significant at *p*-value < 0.001

## Discussion

This study explored the predictors of maternal health service utilization during pregnancy, delivery, and the postnatal period using a multicounty representative survey. The study revealed that the overall maternal health service uptake in Western Africa region remains low, with only 23.8% of women receiving adequate maternal health services. As there were no multicounty studies that measured the level of maternal health service utilization in this manner, we were unable to compare the current prevalence to other studies. The adoption of each service and the overall composite measure of service utilization level varied greatly across countries. These disparities might be due to a complex interaction of socioeconomic (poverty, education, and employment) [41, 42], cultural and religious [28], structural (healthcare infrastructures, access to services, and quality of care) [43, 44], gender-related (power dynamics) [45, 46], and political (conflict and instability) factors [47].

Maternal education, residence, wealth index, parity, media exposure (radio and television), difficulty or distance to access health care, enrolment in health insurance schemes, attitude towards wife beating, and autonomy in decision-making were identified as significant predictors of partial and adequate maternal health service uptake.

The odds of receiving both partial and adequate maternal health services were lower among women residing in rural areas. This was supported by studies conducted in LMIC, Southeast Asia [48], and the SSA region [21, 49]. This could be explained by geographical constraints, as the majority of rural areas tend to be characterized by long distances and tough landscapes, making access to healthcare facilities difficult for pregnant, labouring, and postpartum women. In addition, many rural areas were marked by higher levels of poverty, limited financial opportunities, and inadequate healthcare facilities, accompanied by fewer skilled healthcare providers, all of which may limit the accessibility and quality of maternal health services, resulting in reduced utilization [28]. Furthermore, gender stereotypes and limited access to information are prominent in rural areas [50], which may impede women's mobility and decision-making power, limiting the capacity to seek maternal health services autonomously.

Women's educational status has an essential impact on healthcare utilization, with those who attended primary and above secondary school being more likely to use health services partially and adequately. This finding has been supported by previous multicountry studies in SSA [10, 47, 51], Africa [52], and LMICs [53, 54]. This could be because higher education is often correlated with enhanced awareness and knowledge of health-related issues, especially maternal health. As a result, educated women are more likely to recognize the significance of good prenatal care, skilled birth attendance, and postnatal care for both the mother and the newborn, which may result in better utilization of maternal health services. Furthermore, education is proven to provide women with better job opportunities, greater health literacy, empowerment, and capacity for decision-making, allowing them to comprehend health information, make well-informed choices, and effectively navigate the healthcare system [55–57]. All of these together could contribute to more adequate and timely utilization of maternal health services.

The wealth status of a household has also been identified as a significant predictor of maternal health service utilization. This was supported by a multicountry study conducted in SSA [28], and national studies conducted in India [58], Nepal [59], Afghanistan [60], and Ethiopia [61]. This could be because wealthier women are more likely to have the ability to afford to pay for prenatal care visits and diagnostic tests. In addition, those women are more likely to live in relative proximity to healthcare facilities and have access to reliable transportation [28]. Furthermore, women from higher socioeconomic backgrounds may have greater assets and autonomy to overcome non-medical hurdles such as cultural beliefs, societal norms, and presumptions that could prevent them from accessing maternal health care [62].

Parity was found to have significant connections with the utilization of maternal health care, with primiparous women having a higher likelihood of receiving both partial and adequate health services. A recent multicountry study done among South Asia and Sub-Saharan African countries [63], a systematic review in SSA [64], and national studies conducted in Brazil [65], Bangladesh and Pakistan [66], and Ethiopia [61] were in tandem with this finding. This might be due to several reasons. To begin, as this is their first pregnancy and childbirth, primiparous women might perceive pregnancy and childbirth as a new and challenging experience; they are likely to access health services and guidance on adequate prenatal and postpartum careIn addition, primiparous women are more prone to have physical, social, and psychological issues and inquiries regarding pregnancy, childbirth, and postpartum recovery [67]. This could lead them to engage with healthcare providers more frequently, increasing the overall utilization of maternal health services. Although being a primiparous woman can have a positive association with healthcare utilization in the current study, it's vital to keep in mind that individual experiences differ and that these relationships may not apply to all primiparous women in another settings. So, it is critical to ensure that all pregnant women, regardless of parity, have equitable access to maternal health care in order to foster healthy pregnancies, births, and postpartum experiences.

Covered by Health Insurance (HI) schemes was a significant predictor of maternal healthcare utilization in the current study. Studies conducted in Africa have shown that health insurance improves maternal health services [68–71]. This could be because HI helps women to alleviate the out-pocket expenditure-led financial stresses associated with pregnancy and childbirth-related care, all of which can incur significant costs [68]. In addition, HI schemes often include health education and counseling services which can empower pregnant women with information about proper maternal health service-seeking behaviors during pregnancy and childbirth, leading to a better continuity of care [70].

Autonomy in decision-making was also identified as a significant predictor of adequate maternal health service utilization. This was in tandem with findings from multi-country studies in Africa [37, 46, 72, 73], Indonesia [62], Bangladesh [74], and Ethiopia [75]. This might be because women with high autonomy are more likely to be empowered and have a sense of agency over their own health, control over financial resources, and transportation which can encourage them to actively seek out and use maternal health care to safeguard their own and their unborn child's well-being [73]. In addition, autonomous women often have better access to information about their health, pregnancy, and available healthcare services via media and other channels, which equip them to use tailored maternal health services adequately. Furthermore, autonomy can empower women to challenge societal norms and attitudes that stigmatize or discriminate against certain health practices or services, and this can lead to increased utilization of maternal health services [76]. As a result, it is of the utmost importance to strengthen strategies that promote gender equality, women's empowerment, and increased access to resources, healthcare, and education, to enhance women’s autonomy in the region which has a substantial impact on increasing the uptake of maternal health services.

Having a low attitude towards a wife beating was found to be a significant predictor of adequate maternal health service uptake. This was supported by studies conducted in SSA [46, 77], Bangladesh [78], Indonesia [79], and Ethiopia [80]. A woman's attitude towards wife-beating is regarded to be a proxy for her perception of her position, with a woman who regards such violence as "unjustifiable" becoming aware of her higher entitlement, self-esteem, and ability to utilize maternal reproductive health care [81]. Women who denounce violence against women, including wife beating, were supposed to have greater autonomy in making their own decisions, including the ability to receive adequate maternal health services.

Media exposure to radio and television had a significant role in increasing the likelihood of receiving adequate maternal health services which is supported by studies conducted in SSA countries [44, 51, 68], and Asia [63, 82]. Radio and television are effective channels to raise awareness by conveying information about maternal health, including the importance of prenatal care, safe delivery practices, and postpartum care to an extensive audience [83]. They can help women and families understand the benefits of seeking timely and appropriate maternal health services, leading to increased motivation to utilize these services.

The current study has both strengths and limitations. To begin, prior studies on maternal health service utilization looked at ANC, SDS, and PNC separately. However, the current study used a different approach by creating a composite index of the three maternal health services and presenting it as an outcome variable in a single analytical framework that may demonstrate the quality of service uptake. In addition, as the study was based on the most recent nationally representative data, that were collected through standardized and validated data collection tools and procedures, the findings can be generalized to women across the region. Furthermore, due to the clustering effect of DHS data, a multilevel multinomial logistic regression analysis was employed to offer better parameter estimates, as well as disaggregated information on individual and community-level characteristics, which is essential for developing contextual interventions. On the other hand, the study has some limitations. First, as the responses for each service and predictor were based on self-report, there may be a possibility of social desirability bias. Second, since the data was from a cross-sectional survey, it can be difficult to establish a causal relationship between the outcome of interest and factors. Furthermore, despite we attempted to use the most recent data for each country, the inclusion of data from different time periods could affect the comparability of the results. Because the survey is based on interviews with inquiries regarding past occurrences, recall bias may occur.

## Conclusion

Only around a quarter of women in the region used maternal health services adequately, which is far too low and signals that much more effort needs to be done in the region to improve service utilization and meet the SDGs. Maternal education, residence, wealth index, parity, media exposure (radio and television), enrolment in health insurance schemes, attitude towards wife beating, and autonomy in decision-making were identified as significant predictors of partial and adequate maternal health service uptake. The governments and stakeholders in the healthcare systems should prioritise and implement interventions that increase women's autonomy in order to improve their health-seeking behaviour for maternal health services. Program planners and health-care providers should give due emphasis for those women with no formal education and from low-income families. Governments, non-governmental organisations, and the private sectors have to work together to boost media access. Finally, efforts should be undertaken to increase citizen enrollment in health insurance programmes.

## Data Availability

The data for this study were obtained from the DHS program with a reasonable request. Thus, the one who needs the data supporting the findings of this study can get it in anonymized form from the DHS website at https://www.dhsprogram.com upon reasonable request in the same manner as the authors did.

## Abbreviations

AIC: Akaike’s information criterion
ANC: Antenatal Care
aRRR: Adjusted Relative Risk Ratio
BIC: Bayesian Information Criterion
DHS: Demographic and Health Survey
GSEM: Generalized Structural Equation Modelling
HI: Health Insurance
ICC: Intra Class Correlation Coefficient
LMIC: Low and Middle-income Countries (LMICs)
PCV: Proportional Change in Variance
PNC: Postnatal Care
SDGs: Sustainable Development Goals
SDS: Skilled Delivery Service
SSA: Sub Saharan Africa
VIF: Variance Inflation Factor
